# Host Range of Carp Edema Virus (CEV) during a Natural Mortality Event in a Minnesota Lake and Update of CEV Associated Mortality Events in the USA

**DOI:** 10.3390/v13030400

**Published:** 2021-03-03

**Authors:** Isaiah E. Tolo, Soumesh K. Padhi, Peter J. Hundt, Przemyslaw G. Bajer, Sunil K. Mor, Nicholas B. D. Phelps

**Affiliations:** 1 Minnesota Aquatic Invasive Species Research Center, University of Minnesota, St. Paul, MN 55108-6074, USA; tolo0007@umn.edu (I.E.T.); spadhi@umn.edu (S.K.P.); hundt002@umn.edu (P.J.H.); bajer003@umn.edu (P.G.B.); kumars@umn.edu (S.K.M.); 2 College of Food, Agriculture and Natural Resource Sciences, Department of Fisheries, Wildlife and Conservation Biology, University of Minnesota, St. Paul, MN 55108-6074, USA; 3 College of Veterinary Medicine, Department of Veterinary Population Medicine and Veterinary Diagnostic Laboratory, University of Minnesota, St. Paul, MN 55108-6074, USA

**Keywords:** carp edema virus, emerging wildlife disease, host range

## Abstract

Mass mortality events of common carp (*Cyprinus carpio*, carp) associated with carp edema virus (CEV) alone or in coinfections with koi herpesvirus (KHV), is an emerging issue. Despite recent outbreaks of CEV in wild carp populations, the host range of North American species has not been well studied. To that end, we intensively sampled carp (n = 106) and co-habiting native fish species (n = 5 species; n = 156 total fish) from a CEV-suspect mass-mortality event of carp in a small Minnesota lake (Lake Swartout). Additionally, fecal and regurgitant samples (n = 73 each) from double-crested cormorants (*Phalacrocorax auritus*, DCCO) were sampled to test the potential of DCCO to act as a vector for virus transmission. CEV was confirmed to be widespread in the Lake Swartout carp population during the outbreak with high viral loads and histological confirmation, suggesting that CEV was the cause of the mortality event. There were no detections of CEV in any native fish species; however, DCCO regurgitant and fecal samples were positive for CEV DNA. In addition, three CEV-positive and one CEV + KHV-positive mortality events were confirmed with no observed mortality or morbidity of non-carp species in other lakes. This study provides evidence that CEV infection and disease may be specific to carp during mortality events with mixed-species populations, identifies DCCO as a potential vector for CEV, and further expands the known range of CEV, as well as coinfections with KHV, in North America.

## 1. Introduction

A major cause of mortality of wild and aquaculture fish stocks worldwide is the result of introduced pathogens, primarily due to anthropogenic translocations [1,2,3]. Indeed, the widespread and high-volume trade of common carp (*Cyprinus carpio*, carp), and their ornamental variety, koi, has been associated with the transportation of viral pathogens worldwide with introductions to naïve captive and wild populations [4,5,6]. Not native to the continent, common carp have been introduced throughout North America for over a century. Spring viremia of carp, koi herpesvirus (KHV) and, more recently, carp edema virus (CEV), have been reported in wild carp populations, perhaps co-introduced with the carp populations or introduced with the release of koi [7,8,9,10]. Because common carp are considered invasive and ecologically damaging in North America, commercial interest and general regard for this invasive species is low [11]; however, disease emergence in native fish species that live amidst dense populations of introduced carp remains a concern.

For CEV in particular, the possibility of transmission to native species is not well defined and remains a major research priority [12]. Only a single study has examined the host range of this virus, in which six fishes found in Europe (common bleak, *Alburnus alburnus*; crucian carp, *Carassius carassius*; Prussian carp, *Carassius gibelio*; common roach, *Rutilus rutilus*; tench, *Tinca tinca*; and European perch, *Perca fluviatilis*) were found to be positive for CEV without the presence of clinical signs after cohabitation in a laboratory setting with CEV positive carp [13]. Detection of CEV in CEV-exposed conspecific fishes without the presence of clinical signs may indicate that these species act as vectors for CEV transmission [13]. However, the lack of clinical signs also does not necessarily rule out that infection has occurred, since the Amur wild carp strain did not develop clinical signs after experimental infection with CEV [14]. Naturally occurring mortality events have not previously been associated with mortality of non-carp species, though to our knowledge, there are no studies that report systematic sampling of non-carp species during natural mortality events. Additionally, CEV has only recently been detected in wild populations in North America [8,9], thus native species may have had only minimal opportunities for CEV exposure.

Understanding the significance of CEV in North America is further complicated by reports of coinfections of CEV + KHV in association with mass mortality events of wild carp [9]. Occurrences of CEV + KHV coinfections have also been reported in koi ponds in Germany and China [15,16] and in mass-mortality events of aquaculture carp in Iraq [17] and the Republic of Korea [18]. This recent increase in reports of coinfections underlines the importance of continued surveillance for both viruses during disease outbreak investigations. In this study, we investigate potential transmission of CEV to native species during a naturally occurring carp mortality event and report additional mortality events attributable to CEV and CEV + KHV coinfection.

## 2. Materials and Methods

### 2.1. Mortality Event Investigation at Lake Swartout

A mortality event at Lake Swartout (Wright County, Minnesota, [45.229583, −94.074965]) was reported via the Minnesota Aquatic Invasive Species Research Center’s fish kill reporting tool (http://z.umn.edu/fishkill, accessed on 1 January 2021) on 23 June 2019 by a local resident and subsequently investigated. Surface water temperatures at Lake Swartout was in the range of 24–28 °C during the sampling periods. On June 24, five live adult carp were obtained via boat electrofishing, euthanized by immersion in a solution of 3 mL/L of pure clove oil (90% Eugenol; Velona, Elk Grove Village, IL, USA), and transported on ice to the University of Minnesota on the same day for diagnostic examination. Separate tissue samples of brain, gill, and kidney were aseptically collected for each adult carp and preserved by freezing at −20 °C prior to testing for KHV and CEV by qPCR (described below).

A portion of the brain, gill and kidney tissue was pooled from three individuals for virus isolation in a ~1:10 (weight:volume) suspension of Hank’s Balanced Salt solution (HBSS; Cellgro, VA, USA) with 100 units of penicillin, streptomycin, and fungizone, and stored at 4 °C for 48 h prior to inoculation on cell culture. A portion of gill, kidney, and spleen tissues from a single fish were preserved in 10% formalin and submitted to the University of Minnesota Veterinary Diagnostic Laboratory for histopathological examination. On 28 June 2019, a two-hour transect of the entire perimeter of the lake was performed by boat electrofishing to determine adult carp density and to count dead fish.

**Figure 1:**
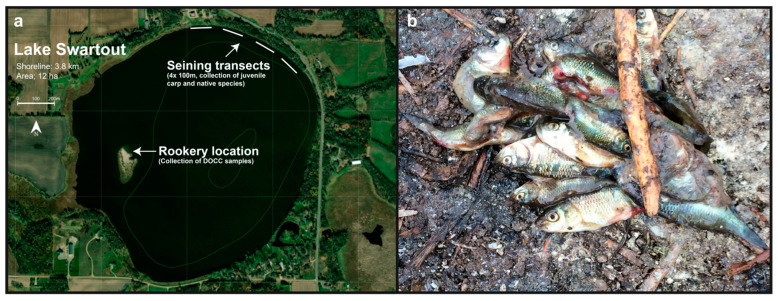
Lake Swartout. Panel (**a**) shows a satellite image of Lake Swartout and surrounding land usage. Seining transects from the collection of juvenile carp and native species and the *Phalacrocorax auritus* (DCCO) rookery site are denoted. Bathymetry contours are in 1.5 m increments. Panel (**b**) shows an image of juvenile carp in a DCCO regurgitant sample.

After detection of CEV was confirmed in initial samples of adult carp from Lake Swartout, additional samples of two adult carp, 99 juvenile carp (<6 cm in standard length; 45 dead and 54 live), and 156 native fishes/amphibians (six dead and 150 live) were collected between 26 June and 10 July 2019 (Table 1). Live juvenile carp and native species were collected using a bag seine in four, 100 m transects performed on 2 July (Figure 1a). We chose a sample size (n) of ~150 live individuals belonging to native species to demonstrate 95% confidence (population level sensitivity, P) of freedom of disease with a design pathogen prevalence (p) of 2% and 99% sensitivity of the diagnostic test (t). Sample size was calculated using the binomial expression; n = log(1 − P)/log(1 − p × t) [19]. During collection, juvenile carp and native species were sorted into separate buckets and euthanized separately by immersion in a solution of 3 mL/L of pure clove oil (90% Eugenol; Velona) and transported on ice to the University of Minnesota and preserved at 4 °C for ~12 h prior to necropsy. Prior to necropsy, the exterior of juvenile carp and native species were rinsed with distilled water. Brain, gill, and kidney tissues were sampled from larger fish (~6 cm or larger) and pooled separately in groups of three fish (e.g., pool 1—brain samples from three fish, pool 2—gill samples from 3 fish, pool 3—kidney samples from three fish). Small fish (<6 cm) were processed as whole fish pooled in groups of 2–5 individuals. All tissues were preserved by freezing at −20 °C prior to testing for CEV and KHV by qPCR.

Additionally, following observation of double-crested Cormorant (*Phalacrocorax auritus*, DCCO) chick carcasses located at a rookery on an island in the middle of the lake (Figure 1a), regurgitant’s containing juvenile carp (Figure 1b) and fecal samples (n = 73 of each) were collected from the shoreline on 28 June, 5 July, and 10 July (Table 1, Figure 1b). Regurgitant and fecal samples were transported to the University of Minnesota on ice and preserved by freezing at −20 °C prior to DNA extraction and testing for CEV and KHV by qPCR.

### 2.2. Additional Mortality Event Investigations

**Figure 2:**
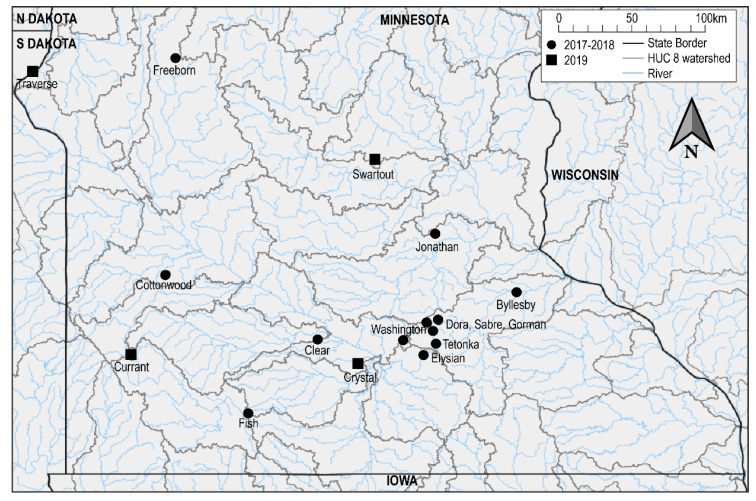
Map of mass mortality events of wild carp in Minnesota associated with CEV or CEV + KHV. Minnesota lakes screened in 2019 (this study) are denoted by black boxes, previously reported mortality events by Padhi et al. 2019 [9], are denoted by black circles. State boundaries, HUC 08 level watersheds, and major rivers are indicated by black, gray, and blue lines, respectively.

We investigated four additional mortality events of common carp in regions where carp mortality events were reported previously [9]: three lakes in Minnesota (Traverse, Crystal, and Currant) and a single mortality event in Pennsylvania (Pymatuning Reservoir) (Table 2, Figure 2). The mortality events were sampled opportunistically based on public reports, consequently the carp carcasses were collected in varying states of decay. Whole carp from the three mortality events in Minnesota were delivered on ice to the University of Minnesota within 24 h of collection from the field and necropsies were immediately performed. Tissue samples (i.e., separate brain, gill and kidney) were aseptically sampled and preserved by freezing at −20 °C prior to testing by qPCR for KHV and CEV. For the mortality event at Pymantuing Reservoir, fresh samples of gill and kidney tissues preserved in 70% ETOH were delivered to the University of Minnesota two days after collection, these tissues were tested for KHV and CEV by qPCR immediately.

Of the four additional mortality events investigated in 2019, two occurred in lakes with previous mortality events associated with KHV or CEV (Table 2). Pymatuning Reservoir in Pennsylvania was previously reported to have a mortality event associated with detection of KHV in 2017, and Lake Currant in Minnesota with coinfection of KHV and CEV in 2018 [9]. Minnesota lakes with mortality events were geographically distant with the closest lakes being 139 km apart (Lakes Crystal and Currant) (Figure 2). None of the lakes were located in the same HUC-8 watershed, but Lake Crystal is located in the same watershed (Middle Minnesota) as Lakes Washington and Clear which had outbreaks associated with KHV in 2017 and 2018, respectively. The event at Pymatuning reservoir occurred earliest and on the largest lake (6915 ha) investigated in this study and had the coldest water temperature of 11.3 °C at the time of sample collection. All investigated mortality events in Minnesota occurred in June, with warmer water temperatures of 18.3–25 °C. The mortality estimates of carp in Lakes Currant, Crystal, and Traverse were all reported by Minnesota Department of Natural Resources (MNDNR) biologists as being in the hundreds of fish and no native fish mortality was observed (Table 2). The mortality of carp in Pymatuning reservoir was reported to be minor by the Pennsylvania Fish and Boat Commission biologists.

### 2.3. Diagnostic Methods for CEV and KHV Detection

All tissue, regurgitant, and fecal samples were homogenized at room temperature in a 1:5 volume of nuclease free water (NFW) (approximately 200 mg of tissue in 1 mL of NFW) then centrifuged for ten minutes at 2380× *g* with 50 uL of the resulting supernatant used for DNA extraction. DNA from adult carp tissues from disease outbreaks were extracted using the Qiagen DNeasy blood & tissue kit (Qiagen, Germantown, MD, USA), following the manufacturer’s protocol for tissues. DNA from juvenile carp and native species survey samples, as well as from DCCO fecal and regurgitant survey samples, were extracted using chelex resin (Sigma, St. Louis, MO, USA) as described by Zida et al. (2019) [20].

We used a Taqman probe-based qPCR using published primers and probes targeting the ORF89 and p4a genes for detection of KHV and CEV, respectively (Table 3) [21,22]. qPCR was performed using a StepOnePlus thermocycler with default settings (Applied Biosystems, Foster City, CA, USA). DNA purifications from all samples were screened for CEV and KHV individually using a PrimeTime**^®^** gene expression master mix kit (Integrated DNA Technologies, Coralville, IA, USA). The reaction mix, containing 400 nM of primers and 250 nM of probe, was subjected to an initial denaturation at 95 °C for 3 min, followed by 40 cycles of denaturation at 95 °C for five sec and annealing at 60 °C for 30 sec. A threshold cycle of 38 was used as a cut off. The standard curve for quantification of CEV and KHV genomes was performed using a laboratory synthesized DNA fragment containing the ORF89 sequence as previously described [9]. The results for virus load are presented as the number of virus copies per 50 uL of tissue homogenate supernatant.

Cell culture methods were performed according to the US Fish and Wildlife Service and American Fisheries Society—Fish Health Section Blue Book (USFWS and AFS-FHS 2016). Briefly, pooled tissues were homogenized in Hank’s Balanced Salt Solution (HBSS; Cellgro, Lincoln, NE, USA) and centrifuged at 2360× g for 15 min. The inoculum was added to 24-well plates with 80% confluent cell cultures of common carp brain cells (CCB) in two dilutions, (1/10 and 1/100) and incubated at 25 °C for 14 days. A blind passage was performed for an additional 14 days if no cytopathic effects (CPE) were observed on the first passage.

Gill, kidney, and spleen tissues from live carp collected on June 24 were examined by light microscopy. Formalin fixed tissues were processed following standard histological methods and stained with hematoxylin and eosin stain.

## 3. Results

### 3.1. Mortality Event Investigation on Lake Swartout

A total of 405 dead adult carp were counted during the boat survey (June 28) and hundreds of dead juvenile carp were observed on the shoreline but were not exhaustively counted. Only two live carp were obtained from two hours of boat electrofishing on June 28, indicating that very few adult carp were left in the lake, however many apparently healthy juvenile carp were observed. Additionally, three dead fathead minnows (*Pimephales promelas*) and three dead leopard frog tadpoles (*Lithobates pipiens*) were observed (June 26) and no other morbidity or mortality of native aquatic species were observed. Mortality of DCCO chicks was also observed on a rookery located on an island in the middle of the lake (Figure 1a) but mortality was later determined by the MNDNR to be the result of infection of DCCO with Virulent Newcastle Disease virus (Minnesota Board of Animal Health, 26 July 2019, https://content.govdelivery.com/accounts/MNBAH/bulletins/254d447, accessed on 1 January 2021).

### 3.2. Investigation of CEV Species Specificity

CEV was detected in all adult and juvenile carp sampled from Lake Swartout (June 24, 26, and 28) and CEV copy numbers were highest in adult carp sampled on June 24 and in dead juvenile carp sampled on June 28 (Table 1). All carp were negative for KHV by qPCR. Tissue samples submitted for virology were negative for any cytopathic effects after the two passages. Histopathology of carp gill tissue from Lake Swartout (collected on June 24) revealed lamellar epithelial hyperplasia and fusion, epithelial sloughing, necrosis, apoptosis, inflammatory infiltrate, and lamellar epithelial cells containing cytoplasmic inclusions, as well as the presence of ectoparasites including monogeneans and *Trichodina* sp. No significant lesions were observed in the kidney or spleen, with the exception of mineralization in kidney tissue.

Despite confirmation of high viral loads of CEV in adult and juvenile carp at the time of native fish and amphibian sampling, CEV was not detected by qPCR in any native species sampled from Lake Swartout, indicating a 95% confidence that the native fish/amphibian population (assuming equal susceptibility among native species) was not infected with CEV at a prevalence of 2% or greater (Table 1). Furthermore, native species mortality was not observed in any of the additional carp mortality events associated with CEV (below). CEV was detected in DCCO regurgitant and fecal sample pools on June 28 and in regurgitant sample pools collected on July 5 (3/12 pools) and 10 (1/12 pools), respectively, but was not detected in fecal samples on these dates.

### 3.3. Additional Mortality Events Investigations

CEV was detected in all 11 carp sampled from the four mortality events and one lake (Currant) was also positive for KHV (2/2 positive) (Table 2). Gill tissues also had the highest viral load when multiple tissues were positive. Gill lesions, including swelling of gill filaments and necrosis of gill tissue, were observed in all fish sampled from Lake Currant. Gross pathology was not available for gill samples from Pymatuning Reservoir (preserved in ETOH) and Lakes Crystal or Traverse (poor post-mortem condition).

## 4. Discussion

In this study, we found CEV in all carp sampled in Lake Swartout, while no CEV was detected in the five native fishes or amphibian species sampled. Assuming that native species screened in this study are equally susceptible to CEV infection, these results indicate that transmission of CEV from common carp to the native species population did not occur or that transmission was rare (i.e., infection of native fishes <2% prevalence). This conclusion is plausible, since native fishes and amphibians were collected at a time and location when CEV exposure was likely. Native fishes and amphibians were collected during a CEV outbreak event when adult and juvenile carp were infected with CEV at a high prevalence and viral load. Also, native species were captured at the same locations as infected juvenile carp.

Though disease associated with CEV has been consistently limited to carp [12], evidence of transmission of CEV to other species during experimental cohabitation with CEV-infected carp has been demonstrated by Matras et al. (2019) [13]. In those experiments, CEV was detected in gill and skin tissues of heterospecific fish species up to 42 days after short periods of cohabitation with CEV-positive carp (12 and 72 h). The lack of CEV detection in alternative vectors in the present study may be due to differences in transmission dynamics in a naturally occurring system compared with experimental enclosures used by Matras et al. (2019) [13]. Additionally, the heterospecific species assemblage in this study did not overlap with that of Matras et al. (2019) [13], which included *Carassius carassius* and *Carassius gibelio*, minnows of the same family (Cyprinidae) as common carp [23]. The species assemblage in our study included only a single species of minnow (family, Leuciscidae), *Pimephales promelas*, that is common in North America and is more distantly related to carp relative to the species included in the study by Matras et al. (2019) [24]. The lack of evidence of CEV transmission to native species in this study indicates that CEV is not a major threat to fishes native to Minnesota, and that CEV transmission by native fish vectors is unlikely in Minnesota lakes with similar species composition. However, additional host susceptibility testing, and wild population screening is necessary to confirm this finding, and to determine if other species are potential vectors of CEV and in what contexts transmission may be possible.

In this study we identified DCCO as a potential vector for CEV, however we do not present evidence of viable CEV obtained from DCCO samples. Transmission via piscivorous birds has been proposed for other viral and bacterial fish pathogens [25,26,27] and recently, KHV DNA was detected in intestinal contents of four species of migratory ducks by Torres-Meza et al. (2020) [28]. Future research in this area should attempt to isolate or demonstrate viable CEV from DCCO or other piscivorous birds. This may be challenging due to the lack of permissible cell-lines for CEV, however in-vivo infection models may be used to provide evidence of viable CEV transmission [14].

The recent detection of CEV + KHV coinfections worldwide [9,15,16,17,18], is an emerging trend. Previously, mass mortality events of wild common carp in North America have been associated primarily with KHV (n = 13) and to a lesser extent with CEV (n = 3) and coinfections of CEV + KHV (n = 4) [8,9]. Additional mortality events reported by Thresher et al. (2018) [10] (n = 17) were also attributed to KHV but testing for CEV was not reported. In the current study, which has continued surveillance of carp mortality events in 2019, mortality events were associated with CEV alone and coinfection of CEV + KHV. This trend highlights the importance of testing for CEV in cases of carp mortality and may indicate that CEV is emerging as a predominant cause of wild carp mortalities in the region, though continued surveillance must be done to confirm this. In areas where carp are valued as a food, recreational, or ornamental fish, this trend of coinfections may represent a significant issue since it will complicate disease outbreak diagnosis and management [15]. Likewise, uncertainty regarding potential impacts of coinfections complicates the evaluation of KHV as a biocontrol tool for controlling invasive populations of carp, as has been proposed by McColl et al. (2018) [29]. Thus, interpreting the trend and implications of increased detection of CEV + KHV coinfections is an important task.

## Figures and Tables

**Table 1:** Carp Edema Virus (CEV) positive survey samples from Lake Swartout.

Date	Water Temp (°C)	Species	No. Individuals (No.Pools)	Disposition, Pooling	Clinical Signs	Tissue Type	CEV Detection	CEV Avg Log Copy No. (SD)
24-Jun	24	*C. carpio (adult)*	5	live, no pooling	gill lesions	brain	5/5	4.84 (0.62)
						gill	5/5	7.24 (0.22)
						kidney	5/5	6.12 (0.04)
28-Jun	26	*C. carpio (adult)*	2	live, no pooling	gill lesions	brain	2/2	3.89 (0.09)
						gill	2/2	4.86 (0.05)
						kidney	1/2	3.99
		*C. carpio (juvenile)*	45 (9)	dead, pools of 5 individuals	skin lesions	whole fish	9/9	7.22 (0.16)
		*P. promelas*	3 (1)	dead, pool of 3 individuals	none	whole fish	0/1	negative
		*L. pipiens*	3 (1)	dead, pool of 3 individuals	none	whole tadpole	0/1	negative
		*P. auritus*	5 (1)	feces, pool of 5	na	feces	1/1	4.39
				regurgitant, pool of 5	na	regurgitant	1/1	4.67
2-Jul	28	*C. carpio (juvenile)*	33 (11)	live, pool of 3 individuals	skin lesions	whole fish	11/11	5.77 (0.74)
		*C. carpio (juvenile)*	21 (7)	live, pool of 3 individuals	none	brain	6/7	4.43 (0.47)
						gill	7/7	4.61 (0.90)
						kidney	6/7	3.91 (0.25)
		*P. promelas*	36 (12)	live, pool of 3 individuals	none	whole fish	0/12	negative
		*P. promelas*	33 (11)	live, pool of 3 individuals	none	brain	0/11	negative
						gill	0/11	negative
						kidney	0/11	negative
		*L. pipiens*	21 (7)	live, pool of 3 individuals	none	brain	0/7	negative
						gill	0/7	negative
						kidney	0/7	negative
		*C. inconstans*	4 (1)	live, pool of 4 individuals	none	whole fish	0/1	negative
		*L. cyanellus*	2 (1)	live, pool of 2 individuals	none	whole fish	0/1	negative
		*L. gibbosus*	54 (18)	live, pool of 3 individuals	none	brain	0/18	negative
						gill	0/18	negative
						kidney	0/18	negative
5-Jul	27	*P. auritus*	36 (12)	feces, pool of 3	na	feces	0/12	negative
				regurgitant, pool of 3	na	regurgitant	3/12	5.77 (0.64)
10-Jul	27	*P. auritus*	36 (12)	feces, pool of 3	na	feces	0/12	negative
				regurgitant, pool of 3	na	regurgitant	1/12	3.53

**Table 2:** Study lakes and screening of wild carp.

Location					Carp Sampling				Virus Load					
Lake Name (County)	Lake Size (ha)	Mortality Event History	Sampling Date	Water Temp (°C)	Estimated Mortality	Disposition (Condition)	Size (cm)	No. Carp	CEV Avg Log Copy No. (SD)			KHV Avg Log Copy No. (SD)		
									Brain	Gill	Kidney	Brain	Gill	Kidney
Pymatuning (Crawford)	6915	KHV, 2017	30-April	11	few	live (good)	nr	5	not tested	5.53 (0.20)	negative	not tested	negative	negative
Currant (Murray)	164	KHV + CEV, 2018	4-June	20	hundreds	live (fair)	45–48	2	5.03 (0.42)	5.64 (0.40)	5.00 (0.07)	3.65 (0.31)	negative	negative
Crystal (Blue Earth)	13	none	7-June	25	hundreds	dead (poor)	nr	2	3.46	4.40	3.37 (0.20)	negative	negative	negative
Traverse (Traverse)	4390	none	12-June	18	hundreds	dead (poor)	50–70	2	negative	4.01 (0.09)	negative	negative	negative	negative
Swartout (Wright)	12	none	24-June	24	405 ^a^	live (excellent)	63–70	5	4.84 (0.62)	7.24 (0.22)	6.12 (0.04)	negative	negative	negative

^a^ Based on transect count of adult carp.

**Table 3:** List of oligonucleotides used during this study.

Primer Name	Target	Primer Sequence (5′-3′)	References	Target Gene (bp-Length)
KHV-86f	KHV	GAC-GCC-GGA-GAC-CTT-GTG	[21]	ORF 89 (78)
KHV-163r		CGG-GTT-GTT-ATT-TTT-GTC-CTT-GTT		
KHV-109p		[TAMRA] CTT-CCT-CTG-CTC-GGC-GAG-CAC-G-[IBRQ]		
CEFAS_qF	CEV	AGT-TTT-GTA-KAT-TGT-AGC-ATT-TCC	[22]	p4a (76)
CEFAS_qR		GAT-TCC-TCA-AGG-AGT-TDC-AGT-AAA		
CEV qProbe1		[FAM]-AGA-GTT-TGT-TTC-TTG-CCA-TAC-AAA-CT-[BHQ1]		

## Data Availability

The datasets generated and/or analyzed during the study are available in the Data Repository for the University of Minnesota (https://doi.org/10.13020/2gcq-pt83).

## References

[1] Gozlan R.E., Peeler E.J., Longshaw M., St-Hilaire S., Feist S.W. (2006). Effect of Microbial Pathogens on the Diversity of Aquatic Populations, Notably in Europe. Microbes Infect..

[2] Peeler E.J., Oidtmann B.C., Midtlyng P.J., Miossec L., Gozlan R.E. (2011). Non-Native Aquatic Animals Introductions Have Driven Disease Emergence in Europe. Biol. Invasions.

[3] Spikmans F., Lemmers P., op den Camp H.J.M., van Haren E., Kappen F., Blaakmeer A., van der Velde G., van Langevelde F., Leuven R.S.E.W., van Alen T.A. (2020). Impact of the Invasive Alien Topmouth Gudgeon (*Pseudorasbora Parva*) and Its Associated Parasite *Sphaerothecum Destruens* on Native Fish Species. Biol. Invasions.

[4] Rodgers C.J., Mohan C.V., Peeler E.J. (2011). The Spread of Pathogens through Trade in Aquatic Animals and Their Products. Rev. Sci. Tech. De L’oie.

[5] Whittington R.J., Chong R. (2007). Global Trade in Ornamental Fish from an Australian Perspective: The Case for Revised Import Risk Analysis and Management Strategies. Prev. Vet. Med..

[6] Girisha S.K., Kushala K.B., Nithin M.S., Puneeth T.G., Naveen Kumar B.T., Vinay T.N., Suresh T., Ajay S.K., Venugopal M.N., Ramesh K.S. (2020). First Report of the Infectious Spleen and Kidney Necrosis Virus (ISKNV) Infection in Ornamental Fishes in India. Transbound. Emerg. Dis..

[7] Phelps N.B.D., Armién A.G., Mor S.K., Goyal S.M., Warg J.V., Bhagyam R., Monahan T. (2012). Spring Viremia of Carp Virus in Minnehaha Creek, Minnesota. J. Aquat. Anim. Health.

[8] Lovy J., Friend S., Al-Hussinee L., Waltzek T. (2018). First Report of Carp Edema Virus in the Mortality of Wild Common Carp *Cyprinus Carpio* in North America. Dis. Aquat. Org..

[9] Padhi S.K., Tolo I., McEachran M., Primus A., Mor S.K., Phelps N.B.D. (2019). Koi Herpesvirus and Carp Oedema Virus: Infections and Coinfections during Mortality Events of Wild Common Carp in the United States. J. Fish Dis..

[10] Thresher R.E., Allman J., Stremick-Thompson L. (2018). Impacts of an Invasive Virus (CyHV-3) on Established Invasive Populations of Common Carp (*Cyprinus Carpio*) in North America. Biol. Invasions.

[11] Klein Z.B., Quist M.C., Miranda L.E., Marron M.M., Steuck M.J., Hansen K.A. (2018). Commercial Fisheries of the Upper Mississippi River: A Century of Sustained Harvest. Fisheries.

[12] Rehman T., Yin L., Latif M.B., Zhou Y., Wang K., Geng Y., Huang X., Chen D., Fang J., Chen Z. (2020). Current Findings on Carp Edema Virus, Control Challenges, and Future Outlook. Aquac. Int..

[13] Matras M., Stachnik M., Borzym E., Maj-Paluch J., Reichert M. (2019). Potential Vector Species of Carp Edema Virus (CEV). J. Fish Dis..

[14] Adamek M., Oschilewski A., Wohlsein P., Jung-Schroers V., Teitge F., Dawson A., Gela D., Piackova V., Kocour M., Adamek J. (2017). Experimental Infections of Different Carp Strains with the Carp Edema Virus (CEV) Give Insights into the Infection Biology of the Virus and Indicate Possible Solutions to Problems Caused by Koi Sleepy Disease (KSD) in Carp Aquaculture. Vet. Res..

[15] Adamek M., Teitge F., Steinhagen D. (2019). Quantitative Diagnostics of Gill Diseases in Common Carp: Not as Simple as It Seems. Dis. Aquat. Org..

[16] Ouyang P., Yang R., Chen J., Wang K., Geng Y., Lai W., Huang X., Chen D., Fang J., Chen Z. (2018). First Detection of Carp Edema Virus in Association with Cyprinid Herpesvirus 3 in Cultured Ornamental Koi, *Cyprinus Carpio* L., in China. Aquaculture.

[17] Toffan A., Marsella A., Abbadi M., Abass S., Al-Adhadh B., Wood G., Stone D.M. (2020). First Detection of Koi Herpesvirus and Carp Oedema Virus in Iraq Associated with a Mass Mortality in Common Carp (*Cyprinus Carpio*). Transbound. Emerg. Dis..

[18] Kim S.W., Giri S.S., Kim S.G., Kwon J., Oh W.T., Park S.C. (2020). Carp Edema Virus and Cyprinid Herpesvirus-3 Coinfection Is Associated with Mass Mortality of Koi (*Cyprinus Carpio Haematopterus*) in the Republic of Korea. Pathogens.

[19] Epitools—Epidemiological Calculators. https://epitools.ausvet.com.au/.

[20] Zida S., Kolia-Diafouka P., Kania D., Sotto A., Foulongne V., Bolloré K., Ouangraoua S., Méda N., Carrère-Kremer S., Van de Perre P. (2019). Combined Testing for Herpes Simplex Virus and *Mycobacterium Tuberculosis* DNA in Cerebrospinal Fluid of Patients with Aseptic Meningitis in Burkina Faso, West Africa. J. Clin. Lab. Anal..

[21] Gilad O., Yun S., Zagmutt-Vergara F., Leutenegger C., Bercovier H., Hedrick R. (2004). Concentrations of a Koi Herpesvirus (KHV) in Tissues of Experimentally-Infected *Cyprinus Carpio* Koi as Assessed by Real-Time TaqMan PCR. Dis. Aquat. Org..

[22] Matras M., Borzym E., Stone D., Way K., Stachnik M., Maj-Paluch J., Palusińska M., Reichert M. (2017). Carp Edema Virus in Polish Aquaculture—Evidence of Significant Sequence Divergence and a New Lineage in Common Carp *Cyprinus Carpio* (L.). J. Fish Dis..

[23] Tan M., Armbruster J.W. (2018). Phylogenetic Classification of Extant Genera of Fishes of the Order Cypriniformes (Teleostei: Ostariophysi). Zootaxa.

[24] Hirt M.V., Arratia G., Chen W.-J., Mayden R.L., Tang K.L., Wood R.M., Simons A.M. (2017). Effects of Gene Choice, Base Composition and Rate Heterogeneity on Inference and Estimates of Divergence Times in Cypriniform Fishes. Biol. J. Linn. Soc..

[25] McAllister P.E., Owens W.J. (1992). Recovery of Infectious Pancreatic Necrosis Virus from the Faeces of Wild Piscivorous Birds. Aquaculture.

[26] Willumsen B. (1989). Birds and Wild Fish as Potential Vectors of *Yersinia ruckeri*. J. Fish Dis..

[27] Taylor P.W. (1992). Fish-Eating Birds as Potential Vectors of *Edwardsiella ictaluri*. J. Aquat. Anim. Health.

[28] Torres-Meza O.A., Loza-Rubio E., Martínez-Maya J.J., García-Espinosa G. (2020). The First Detection of Koi Herpesvirus (Cy HV 3) in Migratory Wild Ducks in North America. J. Aquat. Anim. Health.

[29] McColl K.A., Sunarto A., Neave M.J. (2018). Biocontrol of Carp: More than Just a Herpesvirus. Front. Microbiol..

